# Unravelling B cell heterogeneity: insights into flow cytometry-gated B cells from single-cell multi-omics data

**DOI:** 10.3389/fimmu.2024.1380386

**Published:** 2024-04-18

**Authors:** Jane I. Pernes, Atheer Alsayah, Felicia Tucci, Rachael J. M. Bashford-Rogers

**Affiliations:** ^1^ Department of Biochemistry, University of Oxford, Oxford, United Kingdom; ^2^ Wellcome Centre for Human Genetics, University of Oxford, Oxford, United Kingdom; ^3^ Applied Genomic Technologies Institute, King Abdulaziz City for Science and Technology (KACST), Riyadh, Saudi Arabia; ^4^ Oxford Cancer Centre, University of Oxford, Oxford, United Kingdom

**Keywords:** B cells, atypical B cells, single cell multi-omics, flow cytometry, CITE-seq

## Abstract

**Introduction:**

B cells play a pivotal role in adaptive immunity which has been extensively characterised primarily via flow cytometry-based gating strategies. This study addresses the discrepancies between flow cytometry-defined B cell subsets and their high-confidence molecular signatures using single-cell multi-omics approaches.

**Methods:**

By analysing multi-omics single-cell data from healthy individuals and patients across diseases, we characterised the level and nature of cellular contamination within standard flow cytometric-based gating, resolved some of the ambiguities in the literature surrounding unconventional B cell subsets, and demonstrated the variable effects of flow cytometric-based gating cellular heterogeneity across diseases.

**Results:**

We showed that flow cytometric-defined B cell populations are heterogenous, and the composition varies significantly between disease states thus affecting the implications of functional studies performed on these populations. Importantly, this paper draws caution on findings about B cell selection and function of flow cytometric-sorted populations, and their roles in disease. As a solution, we developed a simple tool to identify additional markers that can be used to increase the purity of flow-cytometric gated immune cell populations based on multi-omics data (*AlliGateR*). Here, we demonstrate that additional non-linear CD20, CD21 and CD24 gating can increase the purity of both naïve and memory populations.

**Discussion:**

These findings underscore the need to reconsider B cell subset definitions within the literature and propose leveraging single-cell multi-omics data for refined characterisation. We show that single-cell multi-omics technologies represent a powerful tool to bridge the gap between surface marker-based annotations and the intricate molecular characteristics of B cell subsets.

## Introduction

B cells are key components of the adaptive immune system, playing pivotal roles in antibody production, immune cell activation and regulation. Flow cytometry has long served as the standard for characterising and gating B cell populations, offering a broad overview of their phenotypic and functional attributes. However, the overly simplistic gating strategies based on a constrained set of surface markers have proven inadequate in capturing the full spectrum of B cell diversity. Single-cell multi-omics, encompassing genomics, transcriptomics, and proteomics, now provides the resolution required to dissect the intricacies and illuminate the functions of B cell populations with an unprecedented level of precision (1, 2), raising questions about the conventional categorisation of B cell populations.

Recent research highlights the limitations of classical flow cytometric-based B cell classifications, emphasising the necessity for a more nuanced understanding of B cell diversity and functionality. This is particularly highlighted in the inconsistencies in flow cytometric gating of specific B cell populations. A key example of ambiguous flow-cytometric gating is with anergic naïve B cells, atypical memory B cells, age-associated B cells, and double-negative B cells. Anergic naïve B cells are a subset of naïve B cells that are associated with autoreactive B cell receptors (BCRs) and have a state of unresponsiveness to antigen stimulation, thus maintaining immune tolerance and preventing autoimmunity (3). These are typically often dysregulated in immune diseases (4), however, are defined differently between studies using different marker combinations, including autoreactive IgMlo naïve B cells and CD19+ IgD+ IgM− CD27- B cells (3–6). Atypical memory B cells represent a heterogeneous population, called as such due to their lack of CD27 or CD21 expression, but with potential features of B cell memory or antigen experience (7). However, the markers defining these populations are not specific for memory B cells and likely to overlap with other B cell populations (7). Studies suggest that alterations in atypical memory B cell subsets may contribute to the dysregulation of immune responses in a range of diseases (8, 9). Age-associated B cells are a population of B cells that increase in frequency with age and exhibit phenotypic and functional alterations, thought to contribute to immunosenescence and increased susceptibility to infections, autoimmune diseases, and decreased vaccine responses in older individuals (10–12). Autoreactive anergic naive B cells (IgM-IgD+), termed B_ND_ cells, have been shown to make up ~2.5% of total B cells and are enriched in autoreactive BCR specificities (3). Double negative B cells (DNB), marked by their CD27 and IgD negativity, have been shown to be elevated in systemic autoimmune diseases such as systemic lupus erythematosus (SLE) and antiphospholipid syndrome (APS), and associated with renal impairment, suggesting a pathogenic role in autoimmunity (13–15). Despite comprising a substantial proportion of B cells, the contribution of DNBs in human health and disease is less well-defined (13–15).

Understanding the roles of these B cell subsets in health and disease is crucial for deciphering their contributions to immune regulation, responses to infections, autoimmune disorders, and age-related changes in the immune system. However, although there are obvious overlaps in the flow-cytometric gating of many of these populations, a systematic understanding of this has not been assessed. Establishing robust gating strategies for these B cell subsets and determining their heterogeneities and relationships is pivotal not only for unravelling their specific functions and interactions within the immune system, but also for their potential roles as biomarkers or therapeutic targets in various pathological conditions. Misclassification or inadequate isolation of these populations could lead to incorrect interpretations of their functional roles, dynamics in disease progression, or responses to therapeutic interventions.

Here, we sought to address the disparities between the conventional flow cytometric-style based annotations of B cell populations and the molecular signatures of individual B cells identified through single-cell multi-omics approaches. By analysing multi-omics single-cell data from healthy individuals and patients across diseases, we characterised the level and nature of cellular contamination within standard flow cytometric-based gating, resolved the ambiguities in the literature surrounding atypical memory cells, and demonstrated the variable effects of flow cytometric-based gating cellular heterogeneity across diseases. Importantly, we showed that flow cytometric-defined B cell populations are heterogenous, and the composition of true naïve, memory and plasmablast B cells from cytometric-defined B cell populations significantly varies between disease states. We characterised the heterogeneity of anergic B cells, age-associated B cells, autoreactive IgMlo naïve B cells, B_ND_ cells, CD21- atypical B cells, and double negative B cells (DNB) and quantified the overlap in gating between multiple studies. Finally, we assessed the variation in cellular impurities in flow cytometric-based gating between disease states. Together, this has implications on functional experiments performed using B cell populations via Fluorescence-Activated Cell Sorting (FACS), where effects between disease states may be driven by differential B cell composition and level of contamination, rather than cell-intrinsic effects. Importantly, this paper draws caution on findings about B cell selection and function of flow cytometric-sorted populations, and their roles in disease. Finally, we offer solutions for identifying improved gating for sorting purer B cell populations through the interrogation of single-cell multi-omics data, and suggest this as a future strategy for functional experiments on immune cell populations. We show that single-cell multi-omics technologies represent a powerful tool to bridge the gap between surface marker-based annotations and the intricate molecular characteristics of B cell subsets, shedding light on the roles of these cells in health and disease and potentially redefining our understanding of immune system function.

## Results

### Direct comparison of classical FACS-style defined B cells with multi-omics-defined annotations

The advent of CITE-Seq technology allows for capturing single cell RNA sequencing along with cell surface protein levels with antibodies conjugated a DNA-barcode, analogous to the fluorophore of flow cytometry antibodies. This allows for the quantitative and qualitative information on surface proteins with available antibodies on a single cell level, with matched RNA-seq, and B cell receptor (BCR) and T cell receptor (TCR) VDJ information (16). This allows us to perform a flow cytometric-style gating of the B cell populations (using CITE-seq) and compare this to the gene expression (GEX) and BCR sequencing (BCR-seq) information. We used data from the COMBAT study (17), which represents a comprehensive single cell multi-omic blood atlas encompassing acute patients with varying COVID-19 severity (18 critical, 20 severe, 12 mild and 12 convalescent), 10 influenza patients, 15 hospitalised sepsis and 10 healthy controls (sampled pre-pandemic). Integrative multi-omics analysis of scRNA-seq, CITE-seq and BCR/TCR-seq allowed for high confidence and quality annotations of B cell, T cell and myeloid populations, as outlined in (17) and characterised in **Supplementary Figure S1**. Briefly, we first performed separate clustering of gene expression, clustering of surface protein expression, and analyses of T and B cell receptor V(D)J sequences [described fully in (17)]. Cell types and subsets were further refined using information from the BCR-seq, CITE-seq and GEX layers for each GEX cluster phenotype led by expert understanding of each immune cell subset, considering a combination of marker genes and transcription factors. Information from all three modalities was used to identify and exclude doublets from downstream analysis. In agreement with the literature, activation markers [CD69, CD80, CD86, CD70, and CD24 (18–20)], cytokines and cytokine receptors [IL-2R, IL-21R, and CXCR3 (21–25)] are elevated in memory and plasmablast populations compared to naïve (20), and IgD, CD21 and CD23 are downregulated (26–29) (**Supplementary Figures S1A–C**). Furthermore, plasmablast/plasma cell-specific transcription factors [IRF4, PRDM1 (BLIMP1), BCL2L1 and XBP1] are observed only in plasmablast populations, whereas early B cell stage TFs [BACH2, PAX5, MCL1 and BCL6 (30, 31)] are down-regulated in plasmablast populations. The GC-stage-specific TF, MYC, is seen highest in memory B cells as expected and decreases upon plasma cell differentiation (32). The transitional and naïve B cells contained no SHM and only unswitched BCRs (IgD/M), whereas the memory and plasmablast populations contained SHM and/or class-switched sequences (**Supplementary Figures S1D, E**). Finally, the level of expression of the heavy chain sequence (nUMIs) and expression of the J-chain was significantly elevated in the plasmablast population compared to the other B cell subsets (**Supplementary Figures S1B, D**) in agreement with elevated production of immunoglobulins (33).

**Figure 1:**
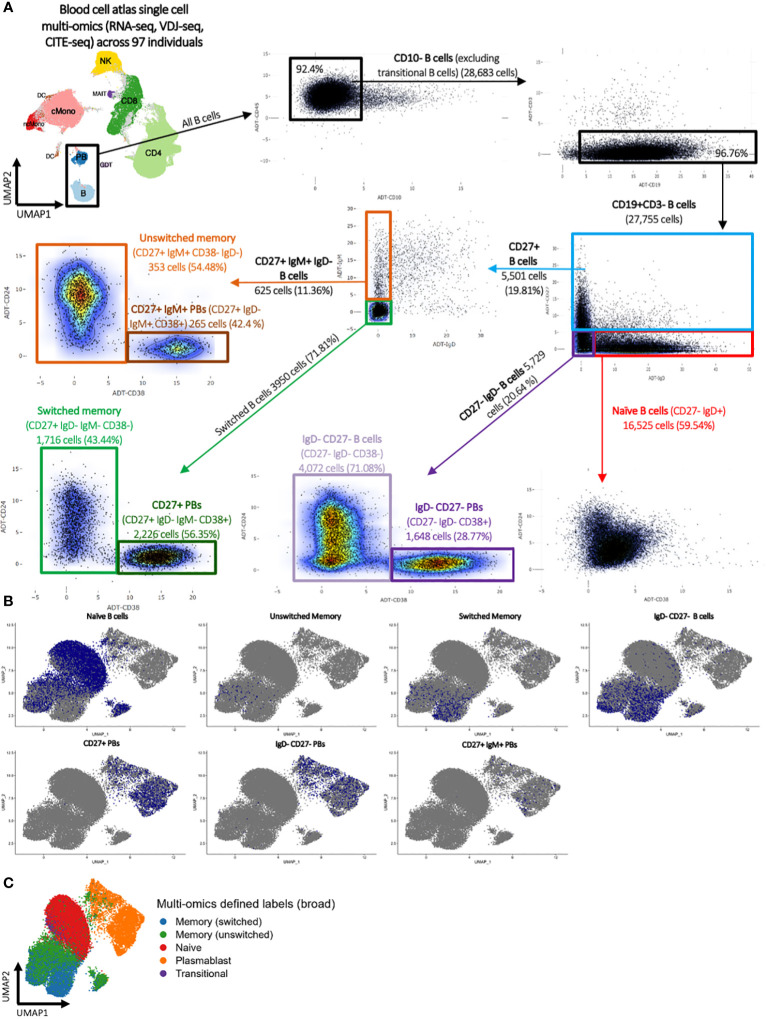
Blood cell atlas single cell multi-omics (RNA-seq, VDJ-seq, CITE-seq) across 97 individuals. **(A)** Classical flow cytometry-style gating strategy using multi-omics CITE-seq levels to define naïve, switched and unswitched memory, IgD- CD27+ B cells, CD27+ plasmablast, CD27+ IgM+ plasmablast, and IgD- CD27+ plasmablast populations. **(B)** UMAP representation of the flow cytometry-style gated B cell populations and **(C)** the multi-omics-informed B cell annotations. The blue dots in panel **(B)** denote the indicated B cells as identified via flow cytometry-style gating, and the grey dots represent the remainder of cells.

Finally, a classical flow cytometric-style gating strategy was performed using multi-omics CITE-seq levels to define naïve, CD27+ IgM- (switched) memory, CD27+ IgM+ (unswitched) memory, IgD- CD27- B cell, CD27+ plasmablast, CD27+ IgM+ plasmablast, and IgD- CD27- plasmablast populations (**Figure 1A**; **Supplementary Table S1**, see methods). These FACS-style gated B cell populations roughly overlaid the high-confidence multi-omics annotations (**Figures 1B, C**).

### Classical FACS-style defined naïve, memory and atypical B cell populations are heterogeneous populations

**Figure 2:**
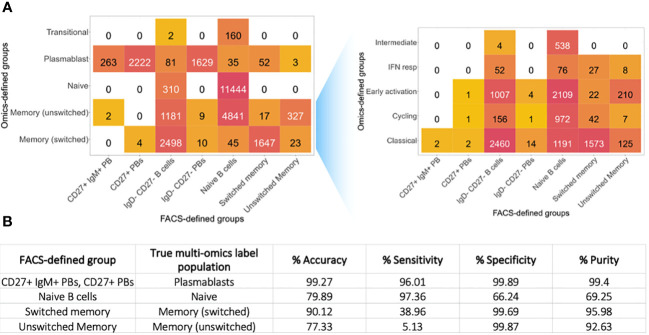
Purity of flow cytometric-style gating. **(A)** Heatmap of the heterogeneity of B cells captured within each standard B cell flow cytometric-style gating for all disease states and health combined. The number represents the number of cells captured with in the corresponding flow-cytometric gate with the corresponding multi-omics label, including memory subtypes. **(B)** Table of the accuracy, sensitivity and specificity of the flow cytometric-style gating to capture target B cell populations, where the true annotations were defined using the multi-omics labelling.

Using both the multi-omics and flow cytometric-style gating approaches for annotating the B cells, we were able to determine the concordance between labelling strategies, and thus characterise the level and nature of cellular contamination within standard flow cytometry gating (**Figure 2A**, cells from all diseases stats and health). While the >99% of flow cytometric-style gated plasmablasts exhibited a plasmablast profile when using all the multi-omic information (multi-omic-annotation), only 69% of flow cytometric-style gated naïve B cells exhibited a multi-omics naïve cell profile. Similarly, only 5.13% of the unswitched memory B cells, as defined by the multi-omics annotation, were captured within the unswitched memory flow cytometric-style gate. Overall, we show the accuracy of flow cytometric-style gating ranged drastically between 77% to >99% depending on the populations of interest (**Figure 2B**). The same trend was observed when considering only cells from healthy individuals (**Supplementary Figure S2**).

**Figure 3:**
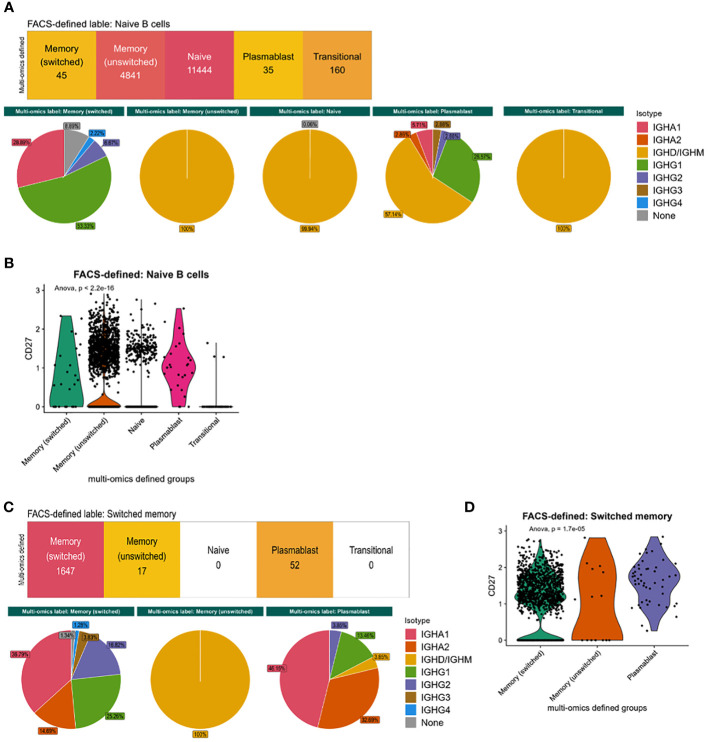
Characteristics of flow cytometric-style gating impurities. **(A)** The isotype distribution of B cells and **(B)** CD27 gene expression within the flow-cytometric-style gated naïve B cells, split by multi-omics annotation. **(C)** The isotype distribution of B cells and **(D)** CD27 gene expression within the flow-cytometric-style gated memory B cells, split by multi-omics annotation. P-values were calculated using ANOVA. This analysis was performed on cells from all disease states and health.

We next explored the cellular heterogeneity within the naïve and memory flow cytometric-style defined populations. 29.5% of flow cytometric-style gated naïve cells were defined as memory B cells via multi-omics information. These different phenotypes had distinct isotype distributions based on immunoglobulin RNA sequence expression (**Figure 3A**) that were significantly different between multi-omics labelled populations (p-values<0.05) and CD27 expression (**Figure 3B**, p-value=2.2e-16)), albeit with low CD27 protein expression (**Figure 1A**). Likewise, 3.0% of the flow cytometric-style gated memory B cells were defined as plasmablasts via multi-omics information. These different phenotypes in the flow cytometric-style gated memory B cells also had distinct isotype distributions based on immunoglobulin RNA sequence expression (**Figure 3C**) that were significantly different between multi-omics labelled populations (p-values<0.05) and CD27 expression (**Figure 3D**, p-value=1.7e-5). Indeed, we show that, while CD27 protein and gene expression is significantly correlated, the correlation is poor across B cell subsets (**Supplementary Figure S2C**). Finally, the flow cytometric-style gating of the switched memory B cells had 90% accuracy, however the gating of unswitched memory B cells was lower (77.3%), with the majority of impurities in this gate consisting of switched memory B cells.

Together this demonstrates that flow cytometric-style gating is successful at enriching particular cell groups such as plasmablasts, however classical naïve and memory B cell gates result in heterogenous B cell populations that can be clearly elucidated considering gene expression and VDJ information. These populations are functionally distinct, with different isotype usages, CD27 expression, and B cell repertoire features.

### Classically-gated anergic, age-associated, autoreactive IgMlo naïve, B_ND_, CD21- atypical, and double negative B cells are highly heterogenous populations

**Figure 4:**
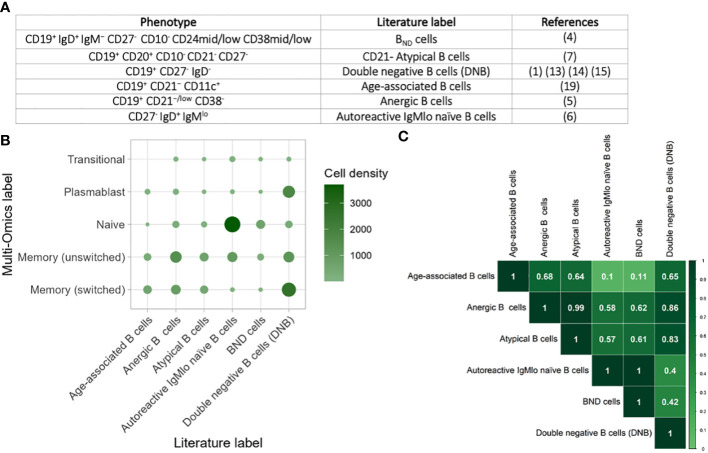
Comparison of atypical B cells from the literature. **(A)** Table of the phenotypic markers used for classifying anergic, age-associated, autoreactive IgMlo naïve, B_ND_, CD21- atypical, and DNB B cells across a subset of studies. **(B)** Heatmap of the heterogeneity of B cells captured within each flow cytometric-style gating from these studies, performed on cells from all disease states and health. The size and colour of each circle represents the number of B cells within each gate that corresponds to the single cell multi-omics label. **(C)** Heatmap of the overlap of B cells captured within gating strategies for the different populations. The values provided represent Jaccard overlap, where a value closer to 1 represents higher overlap between populations.

We next explored the phenotypic heterogeneity of B_ND_ cells (CD19^+^ IgD^+^ IgM^−^ CD27^-^ CD10^-^ CD24mid/low CD38mid/low) (4), CD21- Atypical B cells (CD19^+^ CD20^+^ CD10^-^ CD21^-^ CD27^-^) (7), double negative B cells (DNB) (CD19^+^ CD27^-^ IgD^-^) (1, 13–15), age-associated B cells (CD19^+^ CD21^−^ CD11c^+^) (34), anergic B cells (CD19^+^ CD21^−/low^ CD38^-^) (5), autoreactive IgMlo naïve B cells (CD27^-^ IgD^+^ IgM^lo^) (6) based on FACS gating strategies used in the literature (**Figure 4A**). Using the same gating strategies used in these studies on the CITE-seq values (**Supplementary Figure S3**), we were able to capture each of these populations in the single-cell multi-omics data across health and disease states. Comparison of these populations with the multi-omics annotations revealed significant heterogeneity between these populations (**Figure 4B**). Indeed, age-associated, anergic B cells and CD21- atypical B cells were slightly enriched for unswitched and switched memory B cells, the double negative (DNB) B cells were enriched for switched memory and plasmablasts, whilst IgMlo naïve B cells were enriched for naïve B cells. The B_ND_ cells were not enriched for any specific multi-omics- phenotype, suggesting that CD19^+^ IgD^+^ IgM^−^ CD27^-^ is not a specific gating strategy. Overall, classically-gated anergic, age-associated, autoreactive IgMlo naïve, B_ND_, CD21- atypical, and DNB B cells capture highly heterogenous populations representing diverse gene expression and protein expression patters.

### Significant overlap between anergic, age-associated, autoreactive IgMlo naïve, B_ND_, CD21- atypical, and double negative B cells

To explore this further, we quantified the overlap between classically-gated anergic, age-associated, autoreactive IgMlo naïve, B_ND_, CD21- atypical, and DNB B cells using the Jaccard Index, where a higher value indicates higher levels of overlap between two populations (**Figure 4C**). Whilst some populations were highly distinct with low Jaccard Indices, such as between autoreactive IgMlo naïve B cells and age-associated B cells, we show that there is high overlap between many of the other populations. This is most notable between CD21- atypical B cells (CD19+ CD21− CD11c+) and anergic B cells (CD19+ CD21−/low CD38-) in which the overlap was 0.98 and with the DNB cells (CD19+ CD27- IgD-) in which the overlap was 0.8. Likewise, B_ND_ B cells (CD19+ IgD+ IgM− CD27-) overlap highly with autoreactive IgMlo naïve B cells (overlap = 0.98). Together, this exemplified that these B cell populations are not mutually exclusive, and that functional studies on these populations are often measuring partially overlapping groups of B cells.

### Compositions of classical FACS-style defined B cell populations differs between disease states

**Figure 5:**
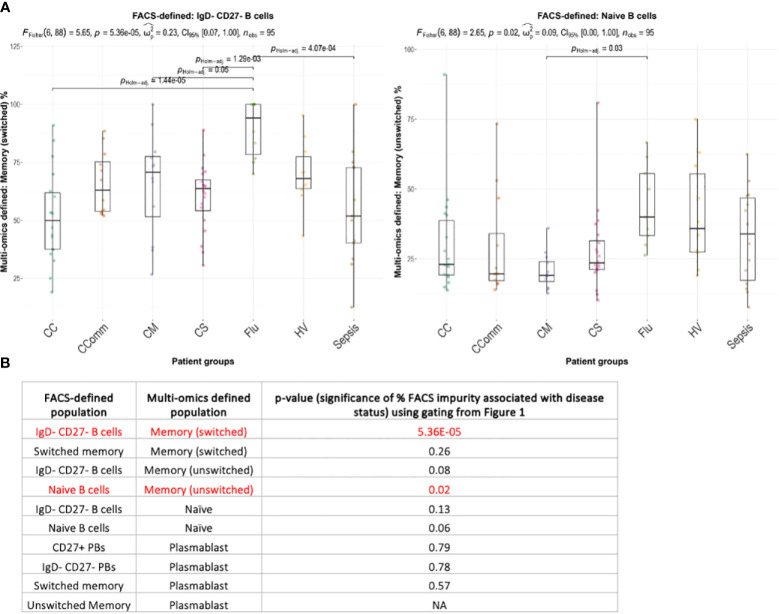
Boxplots of the variation of the proportion of multi-omics-defined B cell populations within flow-cytometric style gating between diseases for **(A)** naïve B cells and IgD- CD27- B cells (showing only those with statistical significance), and **(B)** the corresponding table of significance for all comparisons. Overall p-values of frequencies associating with disease status is provided only for combinations with >3 individuals with non-zero frequencies across each disease state, given at the top of each figure (by ANOVA) and p-values between disease states are provided (by Wilcoxon test using Holm multiple testing correction). Significant values (p-values<0.05) are highlighted in red. CC, Hospitalised COVID-19 (critical); CComm, Healthcare workers COVID-19 (convalescent); CM, COVID-19 (mild); CS, Hospitalised COVID-19 (severe); Flu, Influenza patients; HV, Healthy volunteers; Sepsis, Hospitalised sepsis.

Next, we considered whether the cellular composition of flow cytometric-style gated B cell populations differed between disease states (**Supplementary Table S2**). Using the flow cytometric-style gating of naïve, memory and atypical B cells, we observed significant differences in the proportions of some multi-omics defined populations between disease states (**Figure 5**; **Supplementary Figure S4**, p-values<0.05). Indeed, we show that the proportion of flow cytometric-style gated naïve B cells that have multi-omics profiles of memory unswitched B cells are significantly variable between patient groups, with COVID-19 mild patients with the lowest level of contamination, and influenza patients with the highest level of contamination from unswitched memory switched B cells (**Figures 5A, B**, p-values<0.05). The proportion of flow cytometric-style gated IgD-CD27- B cells comprising multi-omics defined switched memory were significantly associated with disease status (p-values<0.05). Together this demonstrates that the compositions of classical flow cytometric-style defined B cell populations significantly differ between disease state.

### Identification of additional FACS-style markers for homogeneous B cell sorting

**Figure 6:**
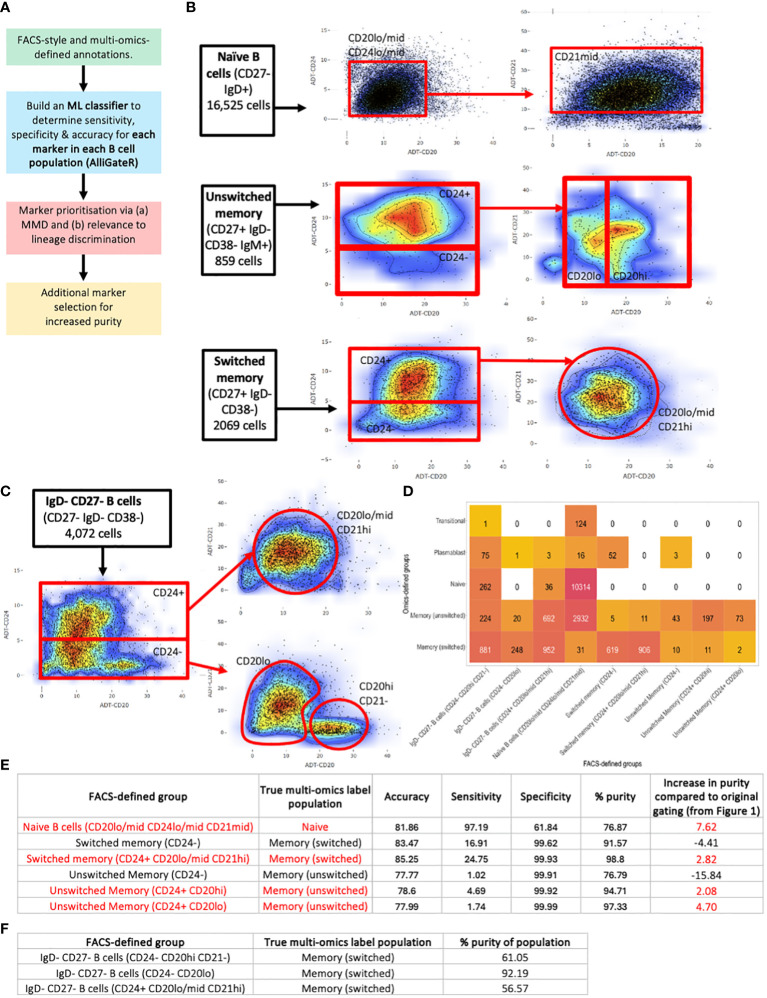
Identification of additional FACS-style markers for homogeneous B cell sorting. **(A)** Strategy for additional marker selection for naïve, unswitched memory, and switched memory B cells, performed on B cells from all disease states and healthy individuals combined. **(B)** Additional gates for (top) naïve B cells, (middle) unswitched memory B cells and (bottom) switched memory B cells. **(C)** Additional gates for IgD- CD27- B cells. **(D)** Heatmap of the heterogeneity of B cells captured using the additional gates. The number represents the number of cells captured with in the corresponding flow-cytometric gate with the corresponding multi-omics label. **(E)** Table of the accuracy, sensitivity, specificity, purity and percentage increase in purity of the flow cytometric-style gating to capture target B cell populations using the additional gates compared to the corresponding multi-omics labels. **(F)** Table of the purity of the flow cytometric-style gating to capture target IgD- CD27- B cell subpopulations using the additional gates compared to the multi-omics label of memory switched.

To overcome the heterogeneity of flow cytometric-style gating of B cell populations, we performed a data-driven analysis of which markers would most appropriately enrich for purer populations of naïve, unswitched memory, switched memory, and IgD- CD27- B cells, that provide a lower level of transcriptional heterogeneity. Here, we considered only the addition of up to 2 additional markers to reflect the constraints of standard FACS sorting or flow-cytometric experiments. It is expected that increasing the number of cell markers will improve the separation of B cell subsets, as compared to gating with a lower number of markers. To achieve this, we performed an unbiased marker selection using a machine learning approach, named *AlliGateR* (All marker enr**i**chment for additional flow-cytometric GATEs for pu**r**er populations) (**Figure 6A**). The Maximum Mean Discrepancy (MMD), which is a measure of dissimilarity between two probability distributions, was used to identify markers that are more adept at discriminating the true populations from impurities identified from the multi-omics data. Finally, the choice of markers needs to be biologically relevant and reflecting lineage definitions, rather than activation status.

For each flow-cytometric gated population, we identified true positive (TP) cells (those that were correctly identified as defined by multi-omics annotations), and false positive (FP) cells (those that were incorrectly identified by flow-cytometric gating but annotated differently by multi-omics analysis), and for each cell surface protein marker, we trained a sigmoidal support vector machine (SVM), and used its predicted annotation (TP or FP) to determine the sensitivity, specificity and accuracy for additional marker selection (**Supplementary Table S3**). From this analysis, a combination of CD20, CD21, and CD24 were found to be the best markers for discriminating TP naïve, unswitched memory, and switched memory B cells from impurities (of which CD24 was already included in the classical gating strategy) (**Supplementary Figure S5A**). However, the multi-omics comparison demonstrated the need for non-linear gates through assessing the highest density of purer B cell populations. These markers were supported by the highly significant differences in protein levels between TP and FP populations (**Supplementary Table S4**). Therefore, additional gating was performed using these markers (**Figures 6B, C**). These additional gates did increase the overall purity of naïve and unswitched and switched memory populations when compared to the original standard gating, but only by between 7.62%, 4.70% and 2.92% respectively. The additional gates for the naïve population (CD20lo/mid CD24lo/mid CD21mid) predominantly reduced the unswitched and switched memory B cell impurities and the plasmablast impurities (by 39.4%, 31.1% and 54.3% respectively, **Supplementary Figure S3B**), which would likely significantly impact the functional readouts of any downstream experiments. The majority of the residual impurities were from transitional B cells.

The additional gates for the switched memory (CD24+ CD20lo/mid CD21hi) removed plasmablast impurities. The unswitched memory B cells were divided into three populations, of which two improved the impurity rate (CD24+ CD20hi and CD24+ CD20lo). The additional CD24- gate for the unswitched and switched memory effectively captured the CD27+ plasmablast impurities (**Supplementary Figure S3B**). We note that additional gating did, however, significantly reduce the number of cells captured within each gate, albeit with lower levels of impurities.

We also investigated whether the IgD- CD27- B cells (also termed double negative B cells, DNB, in the literature, **Figure 3A**) could be subsetted into more homogeneous groups using these markers. Indeed, separating the IgD- CD27- B cells into (a) CD24- CD20hi CD21-, (b) CD24- CD20lo, and (c) CD24+ CD20lo/mid CD21hi, we were able to enrich for specific multi-omics phenotypes. Indeed, 92.19% of the IgD- CD27- [CD24- CD20lo] gated B cells consisted of switched memory B cells as annotated by multi-omics, whereas 41.12%, 56.57%, and 2.14% of the IgD- CD27- [CD24+ CD20lo/mid CD21hi] gated B cells consisted of unswitched memory, switched memory and naïve B cells, respectively, as annotated by multi-omics. Finally, the IgD- CD27- [CD24- CD20hi CD21-] gated B cells inhibited the highest proportion of plasmablasts (5.20%), as annotated by multi-omics, however, the majority of these cells were switched memory B cells (61.05%). Together, we provide a tool to identify additional protein markers that may be used to provide purer populations by flow cytometry, and may be used more generally for other cell types.

Finally, we assessed whether flow-cytometric gated B cell populations with increased purity would reduce the association with disease status. Interestingly, we show that, although the purities of the naïve and memory populations are increased with the additional gates, we showed that there were more associations between disease status and impurity levels of the flow-cytometric gating (**Supplementary Figure S5C**), particularly naïve and switched memory B cells. Overall, we demonstrate a data-driven multi-omics approach to improving experimental purity of B cell populations, and quantify the increased purity of these extra gating approaches. However, this also provides caution on the implications of enumeration and functional readouts of gating strategies when comparing between diseases.

## Discussion

Despite being a long-standing method, conventional gating based on surface markers inadequately captures the extensive diversity and functional roles of B cells. This study focused on elucidating discrepancies between flow cytometry-defined B cell populations and their molecular profiles obtained through single-cell multi-omics analyses. We show that classical flow cytometry-defined populations, particularly naïve, memory and IgD- CD27- B cells, exhibit substantial heterogeneity and inconsistent correlations with their expected phenotypes, as per multi-omics profiles. The discrepancies reveal that conventional gating strategies might inadequately isolate and categorise these subsets, leading to potential misunderstandings of their roles in immune function and disease states. Indeed, we showed that the heterogeneity of these populations is significantly associated with COVID-19 disease status, and this observed variability implies that the cellular composition of flow cytometry-defined B cell populations is disease-specific, potentially influencing functional studies and disease-related investigations. Thus, functional analyses performed on these gating populations would be measuring both intrinsic cellular differences as well as cell-subtype proportion differences (4, 12, 35, 36).

Secondly, this study delineated the complexity and ambiguity in unconventional B cell subsets including anergic, age-associated, autoreactive IgMlo naïve, B_ND_, CD21- atypical, and double negative B cells. We resolved these ambiguities by characterising these subsets using a single-cell multi-omics approach, thereby rectifying misclassifications. Through taking the same gating approach as those used in the original publications, we demonstrated considerable heterogeneity within these populations, each spanning naïve B cells through to memory and plasmablast populations. This inconsistency was best demonstrated by the low overlap between cells gated as anergic within two studies. Instead, the CD21- atypical B cells (CD19+ CD21− CD11c+) from one study overlapped by 98% with anergic B cells (CD19+ CD21−/low CD38-) B cells, whereas the B_ND_ B cells (CD19+ IgD+ IgM− CD27-) from the other study overlapped by 98% with autoreactive IgMlo naïve B cells. This finding highlights the need for a globally agreed consensus on the naming and gating of these populations to build a more consistent understanding of their functional roles in health and disease, reducing the redundancy of cell subtype labelling, and enabling the comparison between independent studies.

Finally, we aimed to address the limitations of conventional flow cytometry gating strategies in defining B cell subsets accurately through using a data-driven approach to suggesting improved gating strategies that improve the purity of the B cell populations sorted. Here, we employed a ML approach (*AlliGateR*) to identify additional gates that may increase purity of any flow-cytometric gates. We have developed this into a generalisable tool that is available of researchers to use on any cell population with the appropriate multi-omics data. With this, we identified three additional non-linear gates using CD20, CD21 and CD24 that were able to increase the subsequent purity of naïve, switched and unswitched memory populations, most significantly for the naïve population (7.62% increase purity). Whilst these additional gates increase the purity of the populations, we finally showed that increases in purity do not translate into reduced association with disease status. Therefore this is a cautionary study showing the implications of enumeration and functional readouts of gating strategies when comparing between diseases.

Overall, we underscore the limitations of conventional flow cytometry-based gating strategies in characterising B cell subsets accurately and disease-associated differences in cellular heterogeneity, which highlight the necessity for refined gating approaches. Ultimately, this work provides a framework for improved B cell characterisation in a data-driven manner, proposing the integration of additional markers for homogeneous sorting to facilitate more precise classification of these subsets and reduced the effect of artefact. This study emphasises the integration single-cell multi-omics technologies as a powerful tool to bridge the gap between surface marker-based annotations and the molecular characteristics of B cell subsets, and immune cells more broadly. Improved characterisation of immune cells may potentially redefine our understanding of immune system function.

## Materials and methods

### Data source

Data was taken from the COMBAT study (17), which included a multi-omic blood atlas encompassing acute patients with varying COVID-19 severity. This data included single cell gene expression, CITE-seq, BCR and TCR VDJ information on matched cells, in which we performed high-confidence annotations of all immune cells with considering all modalities. The full list of CITE-seq markers used are included in **Supplementary Table S1**. This was used as the foundation of this study.

Data pre-processing and annotation using the multi-omics information was performed as described in (17). Briefly, following inspection of the QC metrics, the dataset was filtered to retain cells with ngenes > 300 and pct_mitochondrial < 10%. For the annotation of the immune cell subsets, we used expert immunological knowledge to guide a curated integration of the data from the different modalities (GEX, ADT and VDJ) to identify and label the cell sub-populations present. We first performed separate clustering of gene expression, clustering of surface protein expression, and analyses of T and B cell receptor V(D)J sequences [described fully in (17)]. Cell types and subsets were further refined using information from the repertoire and GEX layers, or in the absence of definitive ADT information were identified by GEX cluster phenotype led by expert understanding of each immune cell subset. Finally, the identified cell types and subsets were further divided by inferred functional state based on targeted assessment of information from all three modalities. For example, cell cycle phase was determined by GEX phenotype, while assignment of B cell maturation status involved use of information from all three modalities (including BCR mutational status). Information from all three modalities was used to identify and exclude doublets from downstream analysis.

### Flow cytometry-style gating

To gate cell population in a flow cytometry-like style, we first identified the most commonly used markers (cell surface markers) to identify each targeted population. *CITEViz* (version 0.1) in R was used to visualise, set thresholds, and gate cells from the original multi-omics dataset based on ADT information. The negative thresholds were based on ADT level densities within populations of cells that are known not to express each marker.

The same methodology was applied to identify and validate markers from the literature. Here we gated B_ND_ cells (CD19^+^ IgD^+^ IgM^−^ CD27^-^ CD10^-^ CD24mid/low CD38mid/low) (4), CD21- Atypical B cells (CD19^+^ CD20^+^ CD10^-^ CD21^-^ CD27^-^) (7), double negative B cells (DNB) (CD19^+^ CD27^-^ IgD^-^) (1, 13–15), age-associated B cells (CD19^+^ CD21^−^ CD11c^+^) (34), anergic B cells (CD19^+^ CD21^−/low^ CD38^-^) (5), autoreactive IgMlo naïve B cells (CD27^-^ IgD^+^ IgM^lo^) (6) based on FACS gating strategies used in the literature (**Figure 4A**; **Supplementary Figure S3**).

### Additional marker prediction

Three methods were used to prioritise additional antibody markers for separating B cell populations. Firstly, we used *FindAllMarkers* function from *Seurat* (version 5.0.1) in R to find differentially expressed markers for each FACS-like cluster. Subsequently, we filtered out any markers with an average log2FC < 2 and adjusted p-value > 0.05. Secondly, we used support vector machine model (svm model) to find the precision, accuracy, sensitivity, recall score, F1 score, false positive and false negative values for each marker in each cluster. Finally, the Maximum Mean Discrepancy (MMD), which is a measure of dissimilarity between two probability distributions, was used. The plasmablast populations (CD27+IgM+ PB, CD27+ PBs, IgD-CD27- PBs) were grouped together for the classification, as they showed a strong overlap with multi-omics plasmablast population. The selected markers were then examined for their effectiveness in distinctly distinguishing their respective cluster from other populations using density map. Population gating with new additional markers was performed as described in the above section, shown in **Figure 6**.

### Statistics

All analysis were conducted using R version (4.2.3). Jensen-Shannon divergence was calculated to measure the similarity between literature-based gating. ANOVA or t-tests was used to find the significance between two groups or more. NS, not significant; *P < 0.05, **P < 0.01, ***P < 0.001, ****P < 0.0001.

## Code availability

Code used in this manuscript is provided in github (https://github.com/AtheerAS/AlliGateR-project).

## Data availability statement

The original contributions presented in the study are included in the article/**Supplementary Material**. Further inquiries can be directed to the corresponding author.

## Ethics statement

The studies involving humans were approved by Sepsis Immunomics REC reference 19/SC/0296; ISARIC WHO Clinical Characterisation Protocol for Severe Emerging Infections REC reference 13/SC/0149. The studies were conducted in accordance with the local legislation and institutional requirements. The participants provided their written informed consent to participate in this study.

## Author contributions

JP: Conceptualization, Data curation, Formal analysis, Funding acquisition, Investigation, Methodology, Project administration, Resources, Software, Supervision, Validation, Visualization, Writing – original draft, Writing – review & editing. AA: Conceptualization, Data curation, Formal analysis, Funding acquisition, Investigation, Methodology, Project administration, Resources, Software, Supervision, Validation, Visualization, Writing – original draft, Writing – review & editing. FT: Conceptualization, Investigation, Supervision, Writing – original draft, Writing – review & editing. RB-R: Conceptualization, Data curation, Formal analysis, Funding acquisition, Investigation, Methodology, Project administration, Resources, Software, Supervision, Validation, Visualization, Writing – original draft, Writing – review & editing.

## Supplementary material

Supplementary Figure 1 Gene expression and VDJ signatures for the single cell multi-omics B cell annotations. **(A)** Gene expression profiles of B cell subpopulations of the top differentially expressed and marker genes. **(B)** The per cell subpopulation (left) somatic hypermutation levels (SHM) and (right) IGHV expression level (nUMIs). Naïve and transitional B cells are marked by low/zero SHM and plasmablasts have the highest IGHV expression. **(C)** The isotype usage percentages across cell types within each cell population. Naïve, transitional B cells and unswitched activated and memory B cells are marked by IGHD/IGHM expression, with only class-switched B cells within the other populations.

Supplementary Figure 2
**(A)** Heatmap of the heterogeneity of B cells captured within each standard B cell flow cytometric-style gating for only healthy individuals. The number represents the number of cells captured with in the corresponding flow-cytometric gate with the corresponding multi-omics label. **(B)** Table of the accuracy, sensitivity and specificity of the flow cytometric-style gating to capture target B cell populations for only healthy individuals, where the true annotations were defined using the multi-omics labelling. **(C)** The correlation between CD27 protein and gene expression within cell subsets. Correlations were performed using Spearman Rank with the corresponding p-values.

Supplementary Figure 3 Gating for the anergic, age-associated, B_ND_, CD21- atypical, and DNB B cells, and autoreactive IgMlo naïve B cells based on flow cytometric gating strategies used in the literature from ().

Supplementary Figure 4 Boxplots of the variation of the proportion of multi-omics-defined B cell populations within flow-cytometric style gating between diseases for naïve B cells, unswitched and switched memory B cells, IgD- CD27- B cells, CD27+ IgM+ plasmablasts, CD27+ plasmablasts and IgD- CD27- plasmablasts. Overall p-values of frequencies associating with disease status is provided at the top of each figure (by ANOVA) and p-values between disease states are provided (by Wilcoxon test using Holm multiple testing correction).

Supplementary Figure 5
**(A)** The distribution of CD20, CD21 and CD24 expression across cells defined by multi-omics. **(B)** Relative change in frequency with additional gates from compared to the gating in (without the additional CD20, CD21 and CD24 gates). The relative change value is between -1 to 0 where -1 represents a complete reduction of a population after the additional gating, and zero represents identical frequencies after the additional gating. **(C)** Table of the variation of the significance (p-value) of association of the percentage of cells labelled via multi-omics-definitions within flow-cytometric style gating between diseases. Overall p-values (calculated by ANOVA) of frequencies associating with disease status is provided only for combinations with >3 individuals with non-zero frequencies across each disease state. Significant values (p-values<0.05) are highlighted in red.
