# Supplementary material for: Unravelling B cell heterogeneity: insights into flow cytometry-gated B cells from single-cell multi-omics data
Source: Front Immunol. 2024 Apr 18;15:1380386. doi: 10.3389/fimmu.2024.1380386 (PMC11067501; doi:10.3389/fimmu.2024.1380386)
Supplement: Supplementary Figure 1 — Gene expression and VDJ signatures for the single cell multi-omics B cell annotations. (A) Gene expression profiles of B cell subpopulations of the top differentially expressed and marker genes. (B) The per cell subpopulation (left) somatic hypermutation levels (SHM) and (right) IGHV expression level (nUMIs). Naïve and transitional B cells are marked by low/zero SHM and plasmablasts have the highest IGHV expression. (C) The isotype usage percentages across cell types within each cell population. Naïve, transitional B cells and unswitched activated and memory B cells are marked by IGHD/IGHM expression, with only class-switched B cells within the other populations. [file Presentation_1.pdf]

Theme: Unswitched Memory B cells in Human Health and Disease

Title: ***Unravelling B cell heterogeneity: Insights into flow cytometry-gated B cells from single-cell multi-omics data***

Jane I. Pernes[*1,2], Atheer Alsayah[*1,3], Felicia Tucci[1,4], Rachael J. M. Bashford-Rogers[1,4]

*Joint first authors.

[1]Department of Biochemistry, South Parks Road, University of Oxford, Oxford, OX1 3QU, UK

[2] Wellcome Centre for Human Genetics, University of Oxford, Oxford, UK

[3]Applied Genomic Technologies Institute, King Abdulaziz City for Science and Technology (KACST), Riyadh, Saudi Arabia.

[4]Oxford Cancer Centre, Oxford, UK

Corresponding author: Rachael Bashford-Rogers (rachael.bashford-rogers@bioch.ox.ac.uk).

Supplemental Figures

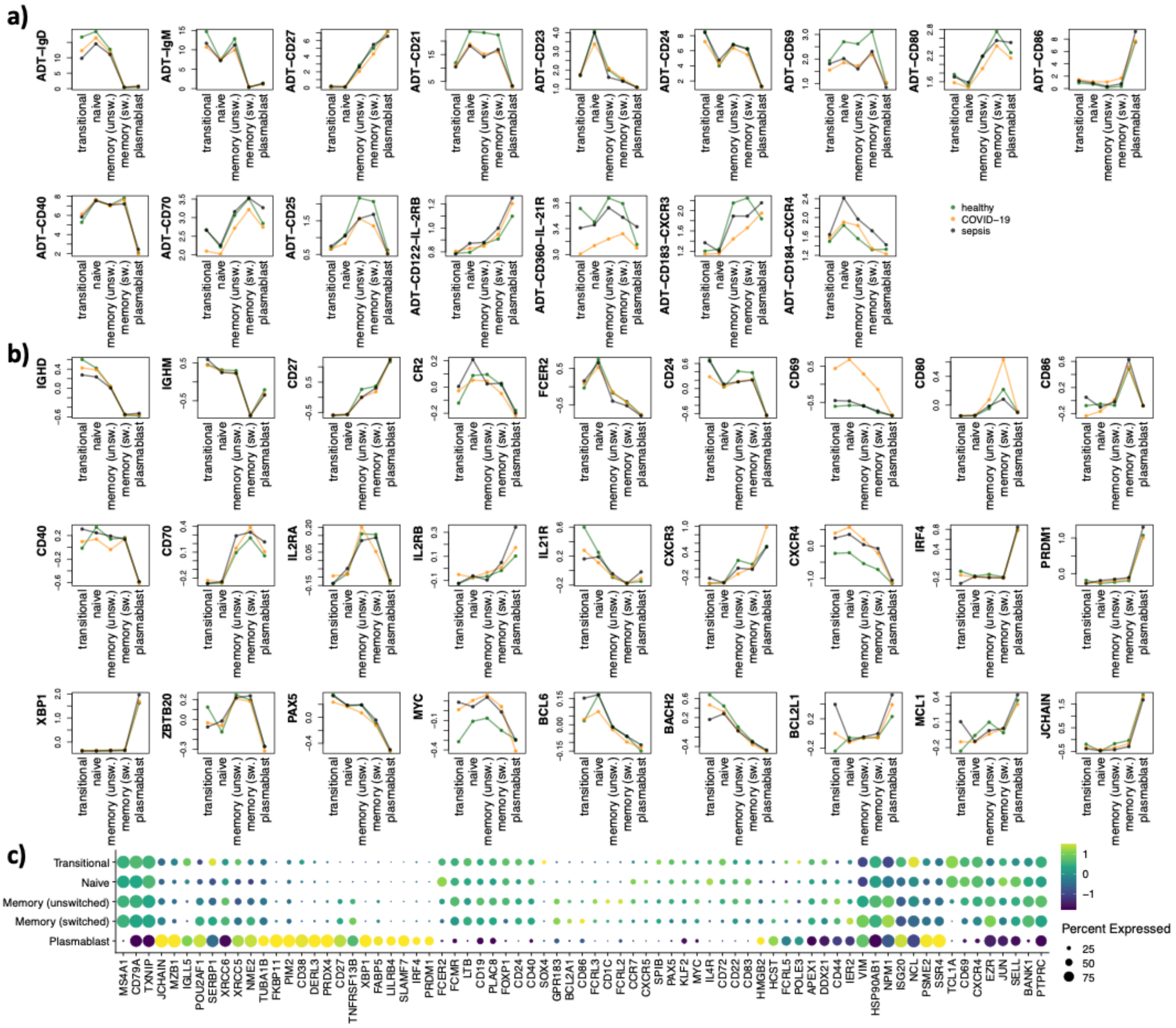

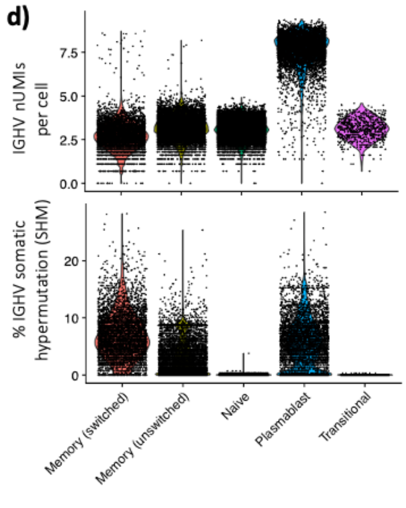

e)

Isotype usage (number of cells)	Multi-omics label				
	Naive	Memory (unswitched)	Memory (switched)	Plasmablast	Transitional
IGHA1	0	0	1597	1589	0
IGHA2	0	0	588	291	0
IGHE	0	0	1	0	0
IGHG1	0	0	1449	2263	0
IGHG2	0	0	554	225	0
IGHG3	0	0	221	118	0
IGHG4	0	0	70	7	0
IGHD/M	13104	7575	0	662	645
None	9	0	70	2	0

Figure S1. Gene expression and VDJ signatures for the single cell multi-omics B cell annotations.

a) Gene expression profiles of B cell subpopulations of the top differentially expressed and marker genes.

b) The per cell subpopulation (left) somatic hypermutation levels (SHM) and (right) IGHV expression level (nUMIs). Naïve and transitional B cells are marked by low/zero SHM and plasmablasts have the highest IGHV expression.

c) The isotype usage percentages across cell types within each cell population. Naïve, transitional B cells amd unswithced activated and memory B cells are marked by IGHD/IGHM expression, with only class-switched B cells within the other populations.

a)

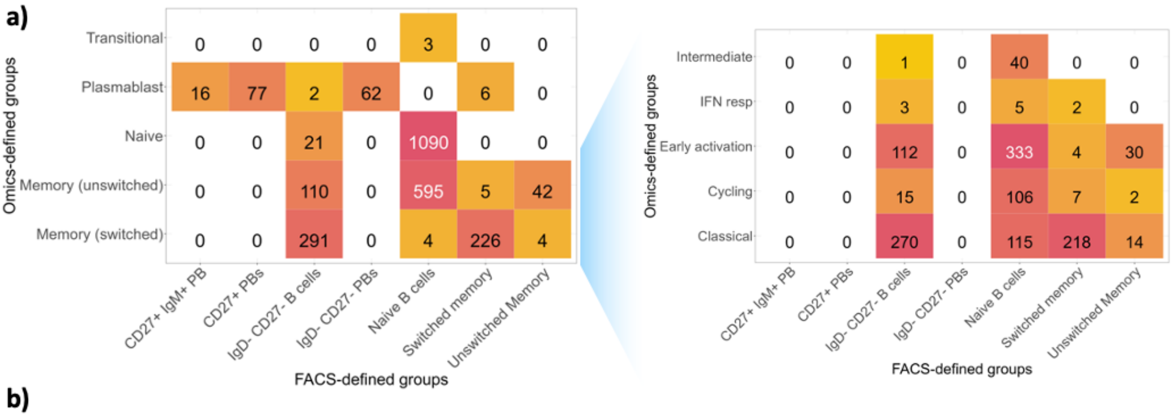

b)

FACS-defined group	True multi-omics label population	% Accuracy	% Sensitivity	% Specificity	% Purity
CD27+ IgM+ PBs, CD27+ PBs, IgD- CD27- PBs	Plasmablast	99.69	95.09	100	100
Naive B cells	Naive	75.61	98.11	58.28	64.42
Switched memory	Memory (switched)	87.86	43.05	99.46	95.36
Unswitched Memory	Memory (unswitched)	72.04	5.59	99.78	91.3

c)

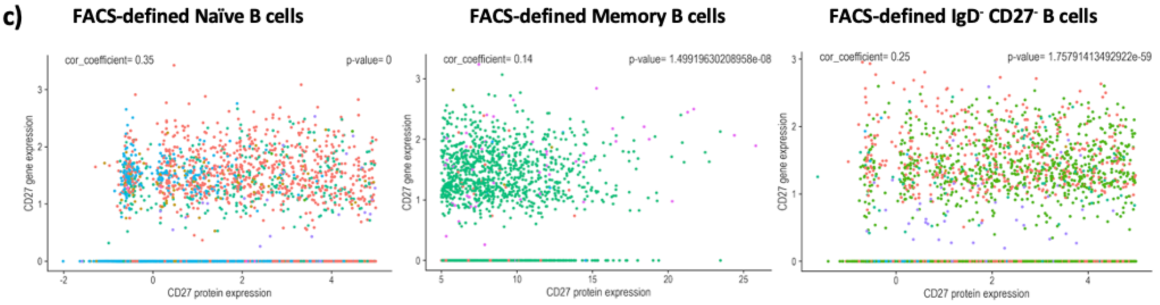

Figure S2.

a) Heatmap of the heterogeneity of B cells captured within each standard B cell flow cytometric-style gating for only healthy individuals. The number represents the number of cells captured with in the corresponding flow-cytometric gate with the corresponding multi-omics label.

b) Table of the accuracy, sensitivity and specificity of the flow cytometric-style gating to capture target B cell populations for only healthy individuals, where the true annotations were defined using the multi-omics labelling.

c) The correlation between CD27 protein and gene expression within cell subsets. Correlations were performed using Spearman Rank with the corresponding p-values.

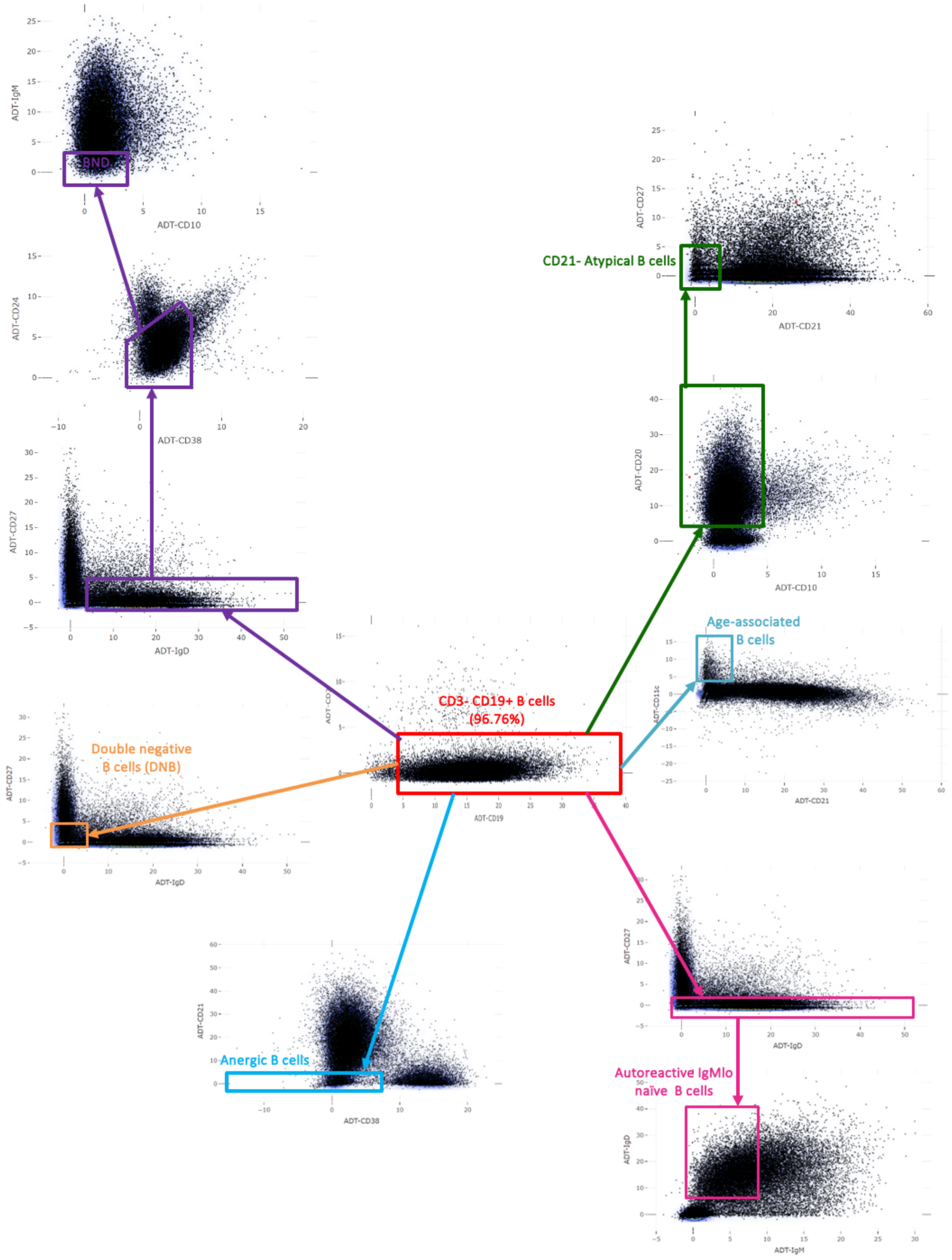

Figure S3. Gating for the anergic, age-associated, B_{ND}, CD21- atypical, and DNB B cells, and autoreactive IgMlo naïve B cells based on flow cytometric gating strategies used in the literature from (**Figure 4a**).

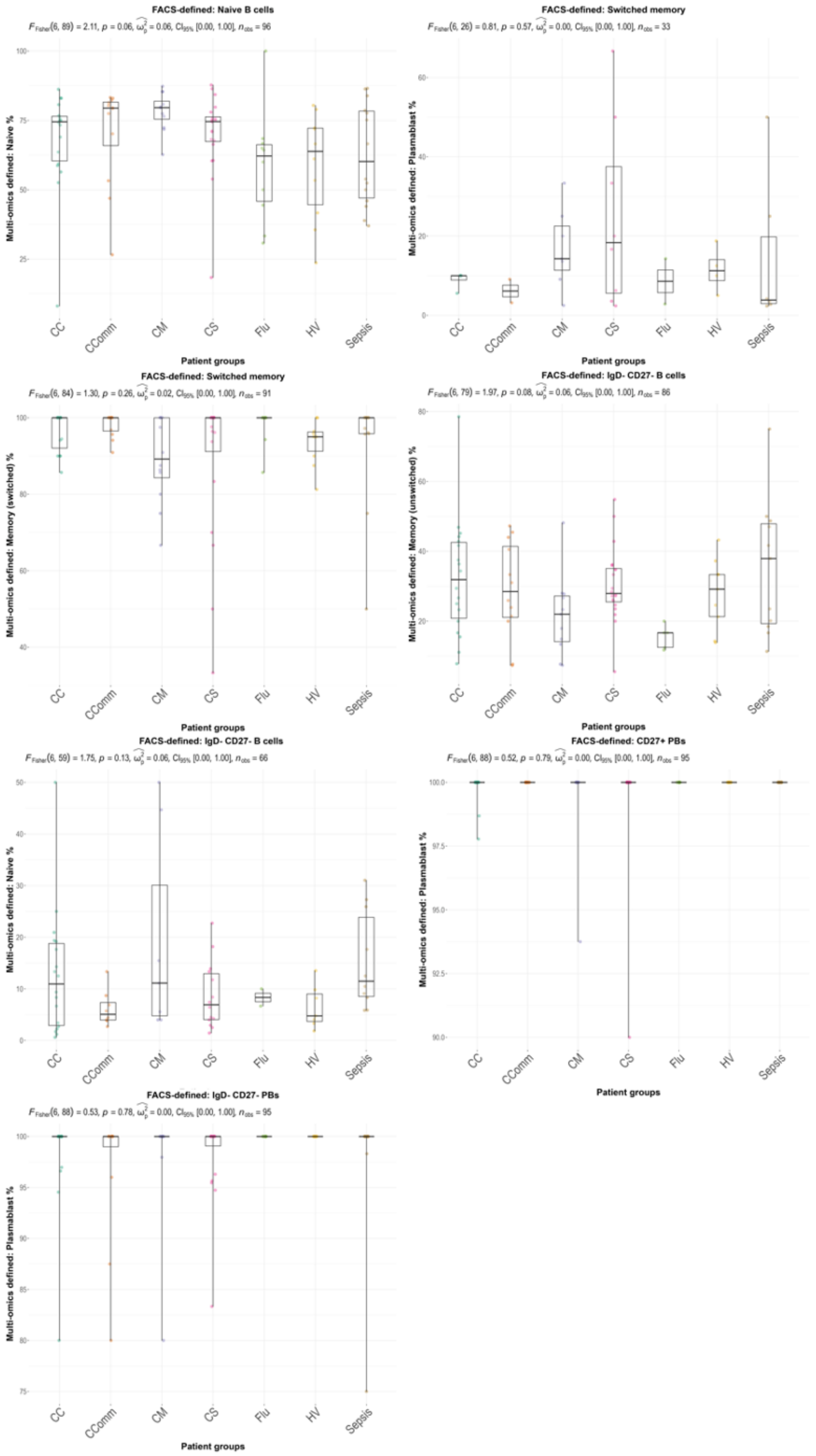

Figure S4. Boxplots of the variation of the proportion of multi-omics-defined B cell populations within flow-cytometric style gating between diseases for naïve B cells, unswitched and switched memory B cells, IgD- CD27- B cells, CD27+ IgM+ plasmablasts, CD27+ plasmablasts and IgD- CD27- plasmablasts.

Overall p-values of frequencies associating with disease status is provided at the top of each figure (by ANOVA) and p-values between disease states are provided (by Wilcoxon test using Holm multiple testing correction).

a)

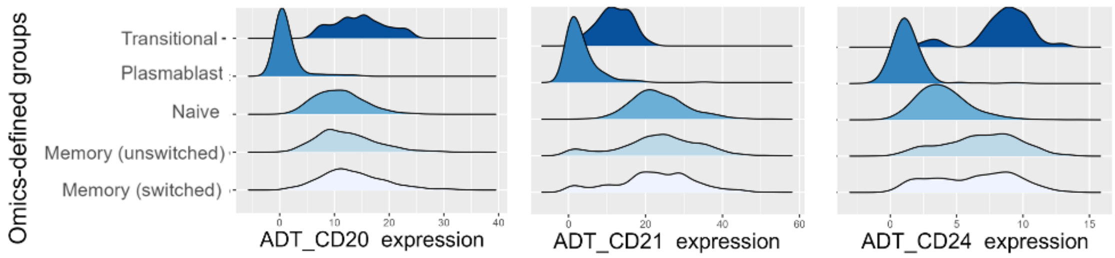

b) ***Relative change in percentage of cells with additional gates 1-3 compared to the gating in Figure 1 (without CD20, CD21 and CD24 gates):***

	**FACS-defined groups**		
**Multi-omics defined labels**	**Naive B cells (CD20lo/mid CD24lo/mid CD21mid)**	**Switched memory (CD24+ CD20lo/mid CD21hi)**	**Unswitched Memory (CD24-)**
Transitional	-0.127	NA	NA
Plasmablast	-0.373	-1.000	-1.000
Naive	-0.052	NA	NA
Memory (unswitched)	-0.246	-0.214	-0.635
Memory (switched)	-0.184	-0.290	-0.840

c)

**FACS-defined population**	**Multi-omics defined population**	**p-value (significance of % FACS impurity associated with disease status) using gating from Figure 6**
IgD-CD27- B cells (CD24-CD20hiCD21-)	Memory (switched)	4.21E-03
IgD-CD27- B cells (CD24-CD20lo)	Memory (switched)	0.83
IgD-CD27- B cells (CD24CD20lomidCD21hi)	Memory (switched)	1.06E-03
Nave B cells (CD20lomidCD24lomidCD21mid)	Memory (switched)	0.02
Switched memory(CD24-)	Memory (switched)	0.24
Switched memory(CD24CD20lomidCD21hi)	Memory (switched)	0.71
Unswitched Memory(CD24CD20lo)	Memory (switched)	NA
IgD-CD27- B cells (CD24-CD20hiCD21-)	Memory (unswitched)	0.91
IgD-CD27- B cells (CD24CD20lomidCD21hi)	Memory (unswitched)	0.11
Nave B cells (CD20lomidCD24lomidCD21mid)	Memory (unswitched)	8.94E-04
Unswitched Memory(CD24CD20lo)	Memory (unswitched)	0.34
IgD-CD27- B cells (CD24-CD20hiCD21-)	Naïve	0.04
Nave B cells (CD20lomidCD24lomidCD21mid)	Naïve	0.07
Nave B cells (CD20lomidCD24lomidCD21mid)	Plasmablast	0.28
Switched memory(CD24-)	Plasmablast	0.57
Unswitched Memory(CD24-)	Plasmablast	NA

Figure S5. a) The distribution of CD20, CD21 and CD24 expression across cells defined by multi-omics. b) Relative change in frequency with additional gates from Figure 6 compared to the gating in Figure 1 (without the additional CD20, CD21 and CD24 gates). The relative change value is between -1 to 0 where -1 represents a complete reduction of a population after the additional gating, and zero represents identical frequencies after the additional gating.

c) Table of the variation of the significance (p-value) of association of the percentage of cells labelled via multi-omics-definitions within flow-cytometric style gating between diseases. Overall p-values (calculated by ANOVA) of frequencies associating with disease status is provided only for combinations with >3 individuals with non-zero frequencies across each disease state. Significant values (p-values<0.05) are highlighted in red.
